# Factors Influencing the Emergence of Heterogeneous Populations of Common Bean (*Phaseolus vulgaris* L.) and Their Potential for Intercropping

**DOI:** 10.3390/plants13081112

**Published:** 2024-04-16

**Authors:** Eva Plestenjak, Vladimir Meglič, Lovro Sinkovič, Barbara Pipan

**Affiliations:** 1 Crop Science Department, Agricultural Institute of Slovenia, Hacquetova Ulica 17, 1000 Ljubljana, Slovenia; vladimir.meglic@kis.si (V.M.); lovro.sinkovic@kis.si (L.S.); barbara.pipan@kis.si (B.P.); 2 Biotechnical Faculty, University of Ljubljana, Jamnikarjeva 101, 1001 Ljubljana, Slovenia

**Keywords:** *Phaseolus vulgaris* L., seed coat colour, seed polymorphism, landraces, intercropping

## Abstract

The common bean is an important legume valued for its protein-rich seeds and its ability to fix nitrogen, making it a key element of crop rotation. In conventional agriculture, the emphasis is on uniformity and genetic purity to optimize crop performance and maximize yields. This is due to both the legal obligations to register varieties and the challenges of implementing breeding programs to create genetically diverse varieties. This paper focuses on the factors that influence the occurrence of heterogeneous common bean populations. The main factors contributing to this diversity have been described, including local adaptations, variable weather conditions, different pollinator species, and intricate interactions between genes controlling seed coat colour. We also discuss the benefits of intercropping common beans for organic farming systems, highlighting the improvement in resistance to diseases, and adverse environmental conditions. This paper contributes to a better understanding of common bean seed heterogeneity and the legal obligation to use heterogeneous populations.

## 1. Common Bean Characteristics

Legumes, the world’s second most important plant family after cereals, are used for both human consumption and as animal feed due to the high protein content of their seeds. They are also known for their root microbial symbioses that lead to the fixation of atmospheric nitrogen and the production of its organic form available for plant growth [1,2]. Growing legumes and non-legumes in the same field can lead to better yields due to nitrogen transfer via the roots. Greater heterogeneity of fields also leads to fewer pest infestations and less use of nitrogen fertilizers and herbicides. Legumes are often used as cover crops to prevent soil erosion [1,3,4]. The common bean (*Phaseolus vulgaris* L.), one of the most important food legumes, is an annual herbaceous plant that is mainly self-pollinated [5,6,7]. It has a small diploid genome (2n = 2x = 22) with 587 Mbp, so it can serve as a model plant for legumes [4,8]. It is mainly cultivated for its edible dry (mature) seeds, shell beans (physiologically mature seeds), and/or green pods, which are high in vitamin C and dietary fibre [9,10]. The dry seeds are an important source of protein, of which phaseolin accounts for about 50% [7]. In addition, studies have shown that phaseolin is a valuable biochemical marker to trace the dispersal pathway of the common bean from its domestication areas to Europe [4,5,11]. The high protein content (between 15% and 45%) and high protein digestibility (almost 80%) is particularly important in developing countries where beans are used as a substitute for animal protein sources [1,10,12]. In addition, the common bean is rich in carbohydrates and has a low glycaemic index compared to other foods, which is important for reducing chronic diseases such as metabolic syndrome. It also contains dietary fibre, micronutrients, vitamins, and phenolic compounds that reduce the incidence of cancer and cardiovascular disease, lower cholesterol levels, regulate diabetes, and have antioxidant and anti-inflammatory effects [10,13].

*Phaseolus vulgaris* L. originated 4 to 6 million years ago in Mesoamerica [3,4,14,15]. Its wild forms are distributed from northern Mexico to northwestern Argentina and are characterized by three gene pools: Mesoamerican, Andean, and Peruvian-Ecuadorian. These gene pools differ from each other based on seed characteristics, such as seed size and weight, as well as the type of phaseolin. Small or medium-sized seeds (with a weight of 100 seeds of less than 25 g to 40 g) and the phaseolin types S (*Sanilac*), B (*Boyacá*), and M (*Middle America*) are characteristics of Mesoamerican origin. Larger seeds (with a weight of 100 seeds of more than 40 g) and the phaseolin types T (*Tendergreen*), H (*Huevo de Huanchaco*), A (*Ayacucho*), J (*Jujuy*), and C (*Contender*), indicate an origin in the Andes. Phaseolin type C may also represent a mixed origin with the Mesoamerican gene pool. The Peru-Ecuador gene pool is characterized by the unique phaseolin type I (*Inca*), which is not present in the other two gene pools [3,4,5,7,11,12,16,17,18,19]. After the formation of these gene pools, domestication occurred, but only in the Andean regions and in Mesoamerica. Domestication of the common bean led to a reduction in genetic diversity and gene expression. A threefold greater reduction was observed in the Mesoamerican gene pool than in the gene pool of the Andean region [3,4,5,6,7,14,16,17,18,20,21]. Differences between domesticated and wild relatives include loss of seed dispersal mechanisms, seed dormancy, photoperiod sensitivity (now almost all common bean varieties are day-neutral, flowering as soon as they are physiologically ready), along with differences in colour, shape, and the size of their edible parts. There are also remarkable differences in the growth habit types. Wild relatives show only indeterminate growth, with the terminal shoot meristem remaining in a vegetative state, i.e., continuously producing a leaf and an inflorescence until senescence. Domesticated bean cultivars may also exhibit a determinate growth habit, generally flowering and maturing early, and the transition of the terminal shoot meristem from the vegetative to the reproductive state results in a terminal inflorescence in the axil of the older leaf primordia [7,14,16,19]. After the discovery of America, the common bean was introduced to new environments and climates, leading to plant adaptation to longer days, cold tolerance, and resistance to pests and diseases. These introductions are proposed as secondary centres of diversification and include Europe, Brazil, Central East and Southeast Africa, and China. In China and Brazil, the Mesoamerican type predominates, while in Africa, both types are equally represented, although they differ from country to country. In Europe, the Andean type predominates, but both types have been introduced [3,4,5,7,17,22]. Mesoamerican beans arrived through Spanish and Portuguese discoveries and were introduced first. Andean beans came to Europe about 20 years later through Pizzaro‘s expeditions in Peru [23]. Although both gene pools were introduced everywhere, hybridization and introgression between the Andean and Mesoamerican gene pools occurred only in Europe [5,7,17,23].

Consumers in different countries and regions prefer certain characteristics of common bean cultivars, depending on the purpose for which they are grown (i.e., grains, pods or combined), growth habit (bush type varieties that do not need support or climbing varieties that need support for growth), length of vegetation, resistance to individual diseases, and colour and size of the seed coat, pods, and other parts of the plant. For consumers of dry bean seeds, the colour and pattern of the seed coat play an important role in the selection process [3,4,5,7,24,25,26]. Genetic and morphological uniformity are necessary not only because of consumer needs, but mainly because of the requirements to be considered in the registration and maintenance of the variety [7,27]. Therefore, common bean breeders strive to produce and maintain genetically uniform commercial varieties with a stable seed coat colour [24,26,28,29,30].

Modern agricultural practices are characterized by relying on a limited number of plant species and cultivate genetically uniform varieties. To avoid crop failure, most of them require crop insurance or other support such as irrigation, nitrogen supplementation, or the use of chemical pesticides. While this strategy makes sense for individual farmers and private breeding companies when weather conditions are stable and government support is available, it carries a significant risk when unpredictable weather events lead to widespread crop failure. Furthermore, despite higher productivity and profitability, they contribute to a decline in biodiversity and increase the risk of genetic erosion [27,31]. In addition, genetic uniformity can be a problem when environmental changes, pest, and disease infestations play a role [32]. Growing a single crop over a large area for several years increases the risk of disease and pest infestation, as there are no other plants or animal species nearby to limit the spread of disease or regulate the occurrence of pests through predation. To avoid losing the entire crop, pesticides and herbicides are used, which can lead to environmental pollution [33,34]. Long-term cultivation of a single crop also reduces the availability of certain nutrients in the soil and degrades soil quality. The loss of nutrients can be replaced by chemical and organic fertilizers, but this is a very expensive process and again, there is the possibility of excess nutrients being released into the environment [1,32,35]. With a growing world population and the associated challenges of climate change, agricultural systems are forced to achieve a sustainable upswing in crop production. This trend can be counteracted by the use of organic breeding strategies and the reintroduction of genetically heterogeneous populations. An even higher level of diversity and stability can be achieved through the legume intercropping [27,31,36].

The background and advantages of heterogeneous common bean populations are still largely unknown. This review therefore provides an overview of the factors that lead to their occurrence, such as the environmental conditions that influence reproductive performance, the outcrossing rate, and the genetic background of seed coat colour, as well as their potential use in organic farming. We also point out the advantages of heterogeneity and greater genetic diversity, e.g., when growing legumes in intercropping systems.

## 2. Seed Heterogeneity Phenomenon in Common Beans

Seeds, a result of sexual reproduction, are an important form in the life cycle of many plants. They enable plants to survive in their habitat, spread into new habitats, and overall ensure the establishment of the next generation. The sowing of seeds and the successful establishment of seedlings is also the crucial first step in global agriculture with annual crops. High quality seed, i.e., high seed viability, purity, and health, is of great importance for the sustainability and profitability of crop production, and is considered a key agronomic trait for all agricultural scales [34,37,38]. Nevertheless, the desire for uniform seed among individual plants of the same species is very high; many higher plants can produce different seed morphs [39,40]. Heterogeneous seeds can result from fluctuations in the environment (differences in light, minerals, temperature, and salinity), spontaneous mutations, and maternal influences (position of the seed within the plant (e.g., its position in the fruit, inflorescence, or overall position of the inflorescence on the mother plant), the age of the mother plant and prolonged flowering periods). These factors can influence gene expression at different levels, e.g., through methylation, histone modification, and other non-coding RNA-mediated processes [40,41,42,43,44]. Variation within an individual also includes differences in seed characteristics, such as differences in seed coat colour, size, shape, dispersal ability, germination, and dormancy. This seed heterogeneity appears to be mainly related to variable environmental conditions and therefore allows plants to adapt and increase the probability of survival of the next generation [39,40,45]. The production of seeds with different morphological traits has been well studied, especially in the angiosperm families *Asteraceae* and *Chenopodiaceae*. Cases of seed heterogeneity in some members of the *Fabaceae* family have also been reported [39,41,43,46,47,48]. Acosta et al. [39] studied the forage legume *Teramnus labialis*, which produces seeds with three different seed coat colours: light brown, brown, and dark brown. Each seed coat colour is associated with a different seed thickness, mass, water content, and, consequently, germination capacity. Seeds with a dark brown coloration absorbed moisture the fastest and showed the highest germination rate [39]. In the study by Dello Jacovo et al. [46], *Lathyrus linifolius* L., a nitrogen-fixing legume, was investigated. They characterized the seeds and found two colour classes of the seed coat, green and brown, which also differed in seed size. Despite their differences, the seeds had a similar germination capacity [46].

**Figure 1:**
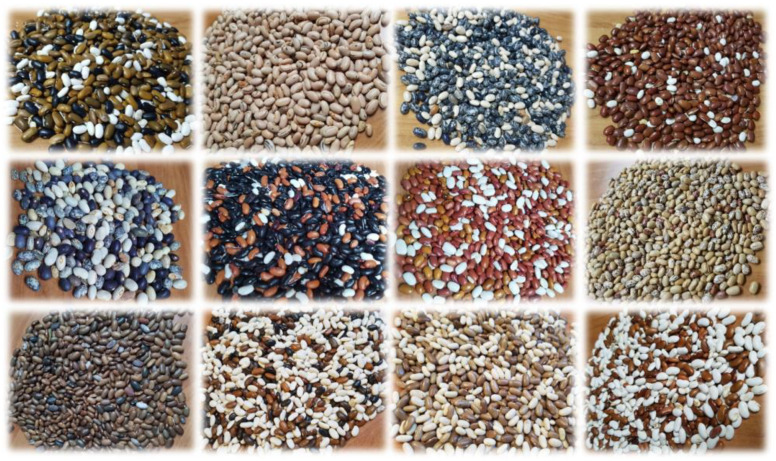
Examples of heterogeneous populations of common bean according to seed coat colour and pattern.

In common beans, the best known form of heterogeneity is that of landraces. These are genetically and phenotypically variable populations of common beans that exhibit rare organoleptic characteristics. They can also show resilience by tolerating a spectrum of biotic and abiotic stress factors, demonstrating their adaptability and robustness under different environmental conditions. Although these populations are under the influence of human selection and environmental stresses, they have existed for a long time, and heterogeneity within these populations appears to be an important adaptive trait (Figure 1) [36,49,50]. Landraces are also not formally improved and are adapted to the specific habitat in which they are grown. They are grown and maintained by local farmers, so there are even different varieties with similar traits but different names in each location [7,25,34,36,50]. They are often cultivated and maintained by older farmers, with little or no support from state or local institutions. Moreover, due to their heterogeneity, they do not meet the requirements for registration as a variety or for commercial cultivation [36].

The commercial production of beans depends on the availability of varieties that meet the needs of producers and consumers. Breeding and the search for new varieties is a continuous process due to the demand for higher productivity, better quality, and greater resistance to pests and abiotic factors [51]. In 1961, the International Union for the Protection of New Varieties of Plants (UPOV) introduced the Plant Variety Rights (PVR), which protects breeders’ investments in the creation of new varieties. Plant Variety Rights (PVR) are based on two tests: Distinctness, Uniformity, and Stability (DUS) and Value for Cultivation and Use (VCU). The DUS test requires that a new variety is distinct from commonly known varieties, maintains the uniformity of the seeds of which the variety is composed, and is stable in different environments. DUS is typically defined by a set of morphological characteristics, sometimes also by isoenzyme electrophoresis and molecular markers. VCU, on the other hand, requires that a new variety shows an improvement in yield, biotic or abiotic resistance, and quality traits [52,53,54,55].

Although uniform varieties are more productive, heterogeneous populations can form a buffer against abiotic and biotic stress. Due to the differences between the plants, not all of them perish under unfavourable environmental conditions, and in the case of disease, it cannot spread to the entire population due to the different resistances. This means that the individual phenotypes within a population compensate each other with their traits and thus maintain the level of adaptability and diversity under different environmental conditions [27,56]. Due to their historical evolutionary background and resilience in stressful environments, landraces in low-input agricultural systems often outperform contemporary varieties in terms of yield [36]. To promote the use of heterogeneous populations, the new EU regulation on organic farming defines the use of diverse plant genetic material, such as organic heterogeneous material (OHM) and organic varieties suitable for organic production. OHM represents a group of plants within a single botanical classification of the lowest known rank that share common phenotypic characteristics and are characterized by a high degree of genetic and phenotypic diversity between individual reproductive units. Therefore, this group of plants is represented by the material as a whole and not by a small number of units; it is not a variety or a mixture of varieties. An organic variety, on the other hand, is a variety characterized by a high degree of genetic and phenotypic diversity between the individual reproductive units and is the result of organic breeding activities [57]. This can increase consumer demand for specific and local products with high quality and special taste. It will also help to preserve landraces of common beans and make them useful for both traditional and molecular breeding programs, as they can contribute valuable alleles [56].

## 3. Background Effects and Mechanisms Reflecting Heterogeneity of Common Bean Seeds

Understanding the factors that contribute to seed heterogeneity is important for plant breeders, farmers, and other stakeholders who rely on high-quality, uniform seed populations for crop production. In autogamous species, of which common bean is one, unfavourable environmental conditions, as well as local adaptations, can influence the increased level of cross-pollination and thus play an important role in maintaining and creating variability [56,58,59]. Klaedtke et al. [58] have shown that historical common bean landraces can change during reproduction in different environments in only three growing seasons. The changes affected both genetic composition and phenotypic traits, and can also be influenced by outcrossing, while other bean varieties were grown nearby during the trials, allowing for potential gene flow between experimental and non-experimental plots [58]. Nevertheless, heterogeneity within the common bean population is a combination of different factors, such as mutations, genetic drifts, environmental conditions affecting outcrossing rates, and different pollinator species with different cross-pollination abilities [56,58,59,60].

### 3.1. Heterogeneity of Seeds as a Result of Outcrossing

The common bean is a predominantly self-pollinating species due to the nature of the flower parts (the stigma is located directly below the anther scars) and the fact that the pollen is released the day before the flower opens [7,61]. Fertilization takes place about eight to nine hours after pollination. However, the common bean does not require pollinators for reproduction; bumblebees and other bees visit its flowers to collect nectar. The different flower colours of the common bean, including purple and pink varieties, attract pollinators and are more common in wild beans [61,62]. During the pollinator’s visit, the stamen column is released by the weight of the insect, which simultaneously leads to self-pollination in the case of an unpollinated flower [63]. When the bee leaves the flower, it can be visited by another pollinator, but the amount of remaining nectar is not visible without probing. This process, in which different pollinators contribute to flower self-pollination and increased yield through physical contact, is called tripping. Even if an insect does not carry pollen, it triggers the fertilization process with the flower’s own pollen [61,63].

Normally, all open flowers are already pollinated, but weather conditions such as high humidity, temperature, and wind can affect the viability of the pollen [7,61]. The male reproductive organs are usually more sensitive to temperatures above 30 °C than the female reproductive organs. It is also important what stage of flower development the high temperatures occur. High night temperatures are most critical during the early reproductive stages of sporogenesis, when pollen abortion and anther indehiscence occur [64]. When stress increases, the female reproductive system can no longer function. Flowers with functional female organs and non-functional male organs due to high temperature stress can be pollinated by foreign pollen when visited by bees or bumblebees, but only if the stigma is receptive to foreign pollen [61,64]. Altered growth conditions can lead to a greater diversity of offspring, which also increases the survival rate [65]. Another factor affecting outcrossing is the location of cultivation, which also depends on the type of pollinator; bumblebees are more attracted to bean flowers than bees [62]. The type of variety and its origin also play a role. Beans from the same gene pool cross more frequently than between different gene pools. Outcrossing also occurs more frequently between Mesoamerican bean gene pools. In North and South America, where there are wild bean varieties, pollen can cross between cultivated and wild relatives [7,61,62]. Farmers also have an influence on cross-pollination through row spacing. The greater the distance between the rows, the lower the frequency of cross-pollination. A distance of 5 m between rows is recommended for certified seed production. Natural hybridization occurs at a row spacing of less than 1 m and varies depending on the bean variety, environment, and amount of pollinators [62,66].

Cytoplasmic male sterility (CMS) is another way of producing heterogeneous progeny. CMS is a widespread phenomenon in higher plants that manifests itself in the inability of a plant to produce viable pollen. In contrast to traditional male sterility, which is caused by nuclear genes, CMS is associated with genetic factors located in the cytoplasm, particularly in the mitochondria. In CMS, the plant’s nuclear genome is normally capable of producing functional pollen, but the presence of certain cytoplasmic genes interferes with this process. The production of viable pollen is a very energy-consuming process, and the number of mitochondria in the cytoplasm must be increased, so a dysfunction associated with the mitochondria can lead to sterility. Depending on the differences in phenotypic expression and developmental mechanisms, CMS can be divided into three groups: structural, sporogenic, and functional CMS. Structural CMS involves the absence of or defects in the development of the anthers or the androecium. In functional CMS, the pollen is unable to germinate. Sporogenic CMS, which is also the most common, is associated with the absence of meiosis and abnormalities in microsporogenesis. It results in underdeveloped anthers and the pollen is absent or sterile [67,68]. CMS is maternally inherited and can be induced by long-distant hybridization (inter- and intra-specific exchange of nuclear and cytoplasmic genomes) or spontaneously. The latter CMS is quite rare, requiring genes for sterility and the non-functional nuclear gene to restore fertility in one genotype [67,69]. In *Phaseolus vulgaris* L., CMS is associated with a unique autonomous transcriptional sequence in the mitochondria; *pvs-orf239*. This sequence encodes the protein ORF239, which is related to microspore development and results in male-sterile lines. It can be lost spontaneously or through the action of the nuclear *Fr* gene, which suppresses the expression of the *pvs* (*Phaseolus vulgaris* L. sterility) DNA sequence and leads to the restoration of fertility. Plants with CMS are usually indistinguishable from other plants based on restorer genes [67,70,71]. In CMS plants without restorer genes and with non-spontaneous reversion, self-fertilization is completely eliminated and the only way to produce offspring is outcrossing. In wild species, induced cytoplasmic male sterility is a mechanism to promote intra-specific gene flow, which contributes to diversity and thus enables survival [7,61,67,69,71].

All these aspects influence the outcrossing rate, which is known to be low [62,66]. Chacón-Sánchez et al. [61] summarised several studies from different countries and in all of them, the outcrossing rate in the common bean varies between 0.004% and 66.8%, with the exception of three studies with values above 39%, such as the study by Wellis et.al [72] in the United States, CA, Irvine, where the outcrossing rate ranged from 11.5% to 66.8%, the study by Brunner and Beaver [73] in Puerto Rico, Mayagueez, where the outcrossing rate ranged from 0.5% to 39.3%, and the study by Gepts et al. [74] in Mexico, Puebla, northern highlands, with 20–55% outcrossing. As mentioned above, several factors influence the outcrossing rate and we can conclude that the average outcrossing is lower, ranging from 0.004% to 14.5%. Heterogeneity can therefore be a consequence of outcrossing [61].

### 3.2. Heterogeneity of Seed as a Consequence of Genetic Interactions

The market classes for dry beans are defined by a number of strict phenotypic characteristics, such as seed size, shape, colour, and pattern. In particular, the colour of the seed coat is an important characteristic for the selection of the common bean and is usually highly variable. Within accessions, seed coat variability can be caused by mutation and subsequent segregation, and can also be explained by epistasis [7,25,28,60,75,76]. The colour of the seed coat is determined by many genes with epistatic interactions that produce primary and secondary colours. The latter appear as different patterns on the seed coat, such as spots, stripes, or different motifs. Among all genes, there is a *P* locus that has been described as the “basic factor” for all genotypes of seed coat colour. It is also responsible for the presence or absence of flower colour [28,75,76]. The *P* gene acts as a regulator of the enzymatic pathway by which the colour pigments are formed and does not produce colour itself. Homozygous recessive *pp* individuals have white seeds and white flowers, independent of the other colour genes involved. Other genes involved in seed coat colour include genes that define colour, such as *C* [*R, Prp*, *Prpi*], *Bic*, *Gy*, *J*, *Prpi-2*, *R-2*, *Z* (together with the basic *P* gene, they produce pale seed coat colours), and those that modify or intensify the colour genes, such as *G*, *B*, *V*, and *Rk* (together with the basic *P* gene and colour genes to intensify colours) (Table 1). In addition to the fully coloured seed coats, there are also partially coloured seed coats that have a coloured zone while the rest of the seed coat is white. The basic factor for fully coloured seeds and flowers is the *T* gene, and partially coloured seed coat patterns require a homozygous *tt* genotype. Other genes that determine the seed coat pattern are listed in Table 1 [28,76,77,78].

The genes for colour, colour modifier, and pattern encode enzymes that catalyse the biosynthesis of pigments that accumulate in the seed coat. As mentioned, the *P* gene is essential for the colour of the seed coat. It acts as a transcription factor that activates the pigment biosynthesis pathway at the primary level [25,28,76,79]. The pigments that determine the colour of the seed coat are flavonoids, which can be divided into three subgroups: flavonols, anthocyanins and proanthocyanidins [25,80,81]. Different possible combinations of flavonoids can result in similar seed coat colours, but, roughly speaking, flavonols represent colourless to pale yellow pigments, anthocyanins represent red, purple, and black pigments, and proanthocyanidins represent colourless pigments that turn brown by darkening after harvest. The environment also influences the flavonoids in the seed coat and affects the composition and concentration of the various pigments [25,75,80].

The *C* locus consists of more genes and is also involved in the inheritance of different seed coat patterns. The motifs include the contrast between the darker pattern and the lighter background (primary colour of the seed coat). In the case of seed coats with a mottling pattern, the seeds of the same plant differ from each other. In heterozygosity (*Cc*), the seed coats are mottled, but in the self-pollinated next generation, 25 % of the seeds always have a darker colour without pattern (*CC*), 25 % have a lighter colour without pattern (*cc*), and 50 % remain the same as the parents and have a mottled pattern of the seed coat (*Cc*). Extreme phenotypically variable seeds on the same plant are also produced by genotype *jj* and can be observed as seeds with differently expressed seed coat colour [28,29,77,82]. The environment can also influence colour expression, particularly with respect to the *B* gene, where seeds are greenish-grey-brown in the greenhouse but more pale grey in the field. The expression of the *rk* gene is also impaired in humid environments. Its expression is weak and unstable; therefore, the colour of the seed coat varies from red to pink [28,83]. Seeds with light background such as pinto, cranberry, and light red kidney bean are affected by postharvest darkening. Seed darkening is strongly dependent on the interaction between genotype and environment [6,84,85]. Genetically, it is determined by the *J* gene (beans with the genotype *jj* do not darken) and the *sd* gene, which determine how quickly the seeds darken. Slow darkening is determined by the *sd* gene (recessive inheritance). Regular darkening is associated with a higher content of proanthocyanidins in the seed coat than in the coat of slowly darkening beans. Storage conditions such as high temperatures, humidity and light lead to the oxidation of proanthocyanidins to visible pigments, which are reflected in a darker or browner seed coat [6,85,86]. The above-mentioned genes, such as *T, P, V, Rk, Sal, Am,* and *Bic,* show a high level of pleiotropic gene expression and control the colour of seed coats and flowers. Most of the genes controlling seed coat pattern (*T, Bip, C, Z,* and *J*) and some intensifying genes (*P, V,* and *Rk*) have multiple alleles with epistatic interactions. Due to the many genes, alleles, and their interactions, different combinations of allelic genes can express the same colour, and many seed coat colours and patterns can be observed within the species [28,79].

**Table 1:** Summary list of genes that define and modify the primary and secondary colour of the seed coat and flower, as well as genes involved in the partial colouration.

Gene Name	Role of Gene
*P* gene ^a,b^	Basic gene for synthesis of transcription factors that enable formation of seed coat and flower colour; has several alleles.
Complex *C* locus ^a,b,c,d,e^	Gene for sulphur-white seed coat colour with several closely linked genes and alleles encoding different seed coat patterns; patterns contrast between darker colour and lighter background.
*R* gene ^a^	Dominant gene for red colour of seed coat (oxblood); a part of *C* locus.
*Prp* gene ^a^	Located within *C* locus and includes *Prp* gene and allele *Prp^i^*; *Prp* gene influences purple-red pod colour; *Prp^i^* codes enhanced anthocyanin expression (IAE) syndrome that results in purple-red flower buds, corollas, pods, petioles, leaf laminae and stems; *Prp^i^* gene in conjunction with other genes also influences colour of seed coat.
*Gy* gene ^a^	Produces a greenish-yellow seed coat colour that is associated with complex *C* locus.
*Chr* gene ^a^	*Chr* gene with *gy* gene ensures a greenish-yellow colouration of hilum ring and corona.
*R-2* gene ^a^	Secondary gene for red seed coat colour.
*Prp^i^-2* gene ^a^	Secondary gene for IAE syndrome, independent of *C* locus; can form two coloured seed coats with other genes.
*J* gene ^a,b,f^	Dominant gene for development of mature seed coat colour; involved in expression of hilum ring colour, darkening effect, and shine; with *t* restrict partly coloured patterns; *L* is a synonym for *j* and *l* for *J.*
*Z* gene (former *D*) ^a^	Affects colour of hilum ring; requires *j* gene for loss of expression; restrict partly coloured patterns with *t* gene; zonal.
*G* gene ^a^	Yellowish-brown factor; has a darkening effect.
*B* gene ^a,c^	Greenish-grey-brown factor.
*V* gene ^a,b^	Purple factor that changes seed coat colour to dark purple or black and flower colour to light pink or white to bishop purple.
*Ane* gene ^a^	With *c^u^* express nebulosus mottling; synonym for vein expression on seed coat and is also associated with *B* gene.
*Bic* gene ^a^	With genotype *TPCJBVRk* express dark olive-brown seed coat colour and bicolour flowers.
*Rk* gene ^a,b,e^	Produces recessive red seed coat colour and influences flower pattern; independent of *C* locus and has multiple alleles.
*Sal* gene ^a,b^	With *v* or *V^w^*^f^ genes produces a red haze on seed coat and salmon red (expressed in a vein pattern) or China rose flowers.
*Am* gene ^a^	Expressed only with the *Sal* gene as oxblood seed coat colour and amaranth (scarlet) flower colour.
*T* locus ^a^	Basic factor for partially coloured seed coats and flowers in homozygous recessive form (*tt*).
*Bip* gene ^a^	With *t* and z genes restrict partly coloured patterns, resulting in bipunctata patterns (*t z bip*); they have multiple alleles.
*Cl* gene ^a^	With *t* and *v* genes express circumlineated zone, a sharp, precipitate-like line that separates each coloured area from white part of seed coat.
*Fib* gene ^a,b^	Restrict partly coloured patterns; fibula arcs.
*Mic* gene ^a^	With *c* and *j* genes express micropyle inpunctata pattern.

^a^ [28]; ^b^ [87]; ^c^ [25]; ^d^ [82]; ^e^ [83]; ^f^ [29].

## 4. Exploiting Heterogeneity and Intercropping Systems in Breeding and Agriculture

Agriculture driven by the pursuit of higher yields has impaired biodiversity. Currently, four main crops, i.e., maize, rice, wheat, and soybeans, occupy the majority of arable land, leading to a decline in biodiversity in the fields [88]. The rapid loss of intraspecific diversity with only a few dominant varieties per crop species is just as worrying as the limited number of cultivated species. Accordingly, it is estimated that the global decline in cultivated species and varieties is mainly due to the selection of improved varieties for intensive agriculture. Elite varieties, valued for their higher yields, have susceptibility problems due to homogeneous genetic and phenotypic traits leading to similar resistance capacity [34,89,90]. The sustainability of high-input agricultural practices is increasingly questioned due to their high reliance on artificial inputs, irrigation and practices that degrade soil fertility and uncultivated ecosystems. This poses a challenge, especially with regard to extreme climatic events and ensuring high and stable yields to feed a constantly growing population. Breeding new varieties has therefore become very difficult, considering all the factors that make it difficult to grow crops and achieve high yields. In the field of agricultural production, the quest for sustainable practices driven by the need for resilience and conversion to organic production methods is paramount [33]. Faced with the challenges posed by climate change, limited resources, and the search for environmentally friendly alternatives, farmers are exploring innovative strategies. One such approach is to increase diversity within plant populations, which is often achieved by growing heterogeneous populations. This strategy focuses on promoting intraspecific diversity within a single plant species to take advantage of inherent genetic variations that can contribute to greater adaptability and resilience. Another option for sustainable agricultural practices is intercropping, an old technique that is regaining importance. In intercropping, two or more plant species are grown simultaneously in the same field. This method takes advantage of interspecific diversity and allows different plants to coexist synergistically [34,89,91,92,93].

### 4.1. Advantages of Heterogeneous Populations and Breeding

Heterogeneous populations represent a buffer potential due to the differences between individuals within a population. Heterogeneity can lead to a stabilization of the production system, as at least some plants within the population can thrive under different conditions such as drought, poor soils, or excessive moisture. It also prevents diseases, pathogens, or pests from becoming established due to the different susceptibility of the plants. The benefits of greater diversity also include suppressing weeds, supporting natural insect predators and pollinators, improving soil quality, and reducing nitrogen leaching into soil and water [94]. Due to natural selection and specific environmental conditions, heterogeneous populations have evolved and continue to evolve differently, with some components dominating in one environment and others in another [93]. Continuous cultivation at different locations leads to the establishment of locally adapted populations that differ in grain yield and plant height [91]. Heterogeneous populations are particularly suitable for organic systems and offer an ecological solution for disease control. In addition, the buffering effect is higher than with uniform material and leads to an increase in yield and yield stability [90,91,93,94]. The use of genetically diverse varieties preserves the evolutionary potential of plant populations. Natural selection can have a lasting effect on heterogeneous populations when they are propagated and replanted on the farm. This allows for the development of local adaptations and evolution over time in response to changing environmental conditions [27].

Until now, the use and production of heterogeneous populations has been limited by legislation and variety registration requirements. The lack of uniformity is also problematic because these populations react differently to agronomic treatments as well as processing and cooking [91]. All the positive aspects of heterogeneity and the need to increase diversity have led to the new EU regulation on organic farming allowing the use and production of heterogeneous material [57]. Long before the new EU regulation, farmers were already using heterogeneity in the form of indigenous varieties for their own needs: landraces. They were bred and developed under the influence of different cultivation methods as an adaptation to a specific location and environmental conditions without being formally improved [34,93]. The use of landraces is an excellent way to increase biodiversity. However, heterogeneity can also be achieved through the manual production of composite crosses and mixtures. Composite crosses (evolutionary/bulk populations) are produced by mixing F1 or F2 seeds from crosses of different varieties. Such composite crosses are then subjected to natural selection over several generations, which increases evolutionary fitness; the latter is highly correlated with important agronomic traits. In general, mixtures are often created by mixing seeds of different pure line varieties; the recommended number is two to five. They have a similar genetic background and are therefore usually produced with the aim of disease resistance. Other characteristics such as maturity time or plant height must be more homogeneous to make them easier to harvest [27]. The mixture of early and late maturing plant varieties, on the other hand, make it possible to achieve a harvest over a longer period of time. They can either be static, i.e., they are formed anew each growing season by mixing the same amount of seed, or dynamic, i.e., they develop over time [93]. Mixtures are usually produced from pure line varieties without specific breeding for mixing ability or performance in heterogeneous populations. However, this may not be ideal for the performance of such a mixture, as success in pure stands does not necessarily indicate success in mixed stands and vice versa. Although varietal mixtures have a slight but significant advantage over the average of their pure line components, it is suggested that mixtures could be optimized by identifying exceptional mixtures that outperform their best components. In addition, the concept of ecological combining ability suggests that selection within very different populations grown under contrasting growing conditions could improve mixing ability. Further research is needed to find acceptable trade-offs between individual competitiveness [27].

Like other plants used in agriculture, the common bean is subject to adaptation to different environments, breeding methods, and cropping systems, and all these factors have led to existing genetic diversity [5,16,36]. In addition to landraces, bean mixtures have been known for a long time, especially in regions in Africa, South America, Nepal, and Brazil [95,96]. Such practices are particularly widespread among smallholder farmers. Mixed crops consist of two to thirty varieties, and such diversity is positively associated with greater yield stability, reduced spread and development of anthracnose and bean fly infestation [96]. However, further and more extensive trials are needed to assess stability, identify the factors influencing yield variability, understand the mechanisms in mixed cropping systems, and investigate specific variety combinations. Trials in different market classes and under real growing conditions are crucial for wider acceptance by bean producers.

### 4.2. Intercropping Systems

Intercropping is an old agricultural practice in which two or more plant species are grown simultaneously in one field. In contrast to monoculture, in which a single plant dominates, intercropping utilizes the complementary interactions between different plant species [97,98]. The individual plants have a complementary plant architecture that enables a more efficient use of sunlight and space. A different spatial organization of roots below ground enables the use of different soil layers, leading to a more efficient use of soil nutrients, water resources and space. The synergistic relationships between co-cultivated species can also lead to better yields, reduced susceptibility to pests and diseases, less weed infestation, and greater resilience to environmental fluctuations. Greater plant diversity provides incentives for a greater diversity of other organisms, such as the presence of different pollinators and microorganisms, which positively influence pollination and biological activities in the soil [33,92,99,100,101]. Intercropping is a common agricultural practice, especially in regions with organic farming, subsistence farming, and limited mechanization. It is widely used by farmers who practice labour-intensive, small-scale, low-input agriculture, as it prevents the risk of total crop failure [1,92,99,100,102].

There are various ways of growing crops together, the most important of which is intercropping. The plants are planted at different times so that only part of their life cycle overlaps and potential competition between the plants is reduced. The first plant can therefore grow and develop its aerial and root system. When the second plant is planted, competition may only be present for a short time and when the first plant is harvested, the second can maximize its growth [92,103]. Depending on how the plants are arranged, there are three types of intercropping: mixed cropping, strip cropping, or row cropping. In mixed cropping, two or more crops are grown simultaneously without adhering to a specific spatial arrangement. The plants are harvested together, and the seed is later separated. Due to the non-specific arrangement, it is difficult to establish an appropriate relationship between the plants, as the interaction between the plants is maximized and can lead to competition. This type of arrangement is primarily used for animal feed and provides higher nutritional value, especially more protein in legume intercrops [102]. A cleaner form with less interaction between crops is strip cropping. Crops are grown in parallel strips, each consisting of several rows. Since interspecific interactions such as shading and wind breakage are relatively limited to the edges, there are fewer interspecific interactions and consequently less competition below ground. This composition also allows the use of machinery that enables separate harvesting [102,104]. The third arrangement is row intercropping, which is similar to strip intercropping. The only difference is that at least one of the crops is grown in one or two rows. It is particularly suitable for non-mechanized sowing and harvesting. Row cropping is still used on smaller farms in developing countries [102]. In all intercropping arrangements, it is important that the crops have enough space to cooperate and not compete. Greater plant diversity in the field leads to more efficient use of light, water and nutrients, but we must not forget that crop production is determined by the absence of limiting factors [102,105].

An important aspect in the optimization of intercropping systems is the strategic integration of legumes. They play an important role in improving soil fertility due to their unique ability to fix atmospheric nitrogen in their root nodules through a symbiotic relationship with nitrogen-fixing bacteria. The nitrogen is released from the nodules into the soil where it can be utilized by other nearby plants. In this way, the plants can thrive without relying on an external source of nitrogen, reducing the need for synthetic fertilizers. This not only promotes sustainable agricultural practices, but also helps protect the environment by minimizing nitrogen runoff [1,101]. The most common by-product of nitrogen fixation is soil acidification, which increases the availability of inorganic phosphorus [33,99,106]. *Rhizobia*, the genus of microorganisms that enable nitrogen fixation, are also associated with bioremediation behaviour, such as the removal of various pollutants from the soil. Legumes also have a positive effect on the physical properties of the soil and increase moisture retention [1,4].

All over the world, various legumes are grown in rotation with various non-legume crops. The best known and most widespread use of legumes is in the cultivation of animal feed. Mixtures include grasses and various species of clover (*Trifolium* spp.) and alfalfa (*Medicago sativa* L.), as they can produce higher biomass under different climatic conditions [1,33,101]. Diverse perennial mixtures offer higher yields and higher protein and fibre content. By including other herbaceous species, they improve the nutritional value of the forage by providing additional minerals. In some cases, forage mixtures are also combinations of peas (*Pisum sativum* L.) and cereals (oat—*Avena sativa* L., barley—*Hordeum vulgare* L. and wheat—*Triticum aestivum* L.), or faba bean (*Vicia faba* L.) and maize (*Zea mays* L.). Similar to perennial intercropping systems, annual systems are also associated with a better protein content, higher yields, and a higher relative feed value [101]. The use of legumes such as lupins (*Lupinus angustifolius* L.), vetches (*Vicia genera* and *Vicia sativa* L.), and clover (*Trifolium* spp.) as cover crops reduces weed infestation due to light competition and also reduces water and wind erosion [1]. In boreal-nemoral regions with longer winters, leaching of nitrogen and phosphorus is also a problem. The use of cover crops is therefore important to “capture” nutrients during autumn growth and make them available in the spring after decomposition [92,101].

Grain legumes that are commonly grown as food crops in intercropping include common bean (*Phaseolus vulgaris* L.), faba bean (*Vicia faba* L.), pea (*Pisum sativum* L.), cowpea (*Vigna unguiculata* L.), and chickpea (*Cicer arietinum* L.) [1]. They can be intercropped with cereals, and such combinations are a good solution due to the high nitrogen requirements of cereals. The result of such combinations is a higher protein concentration than when cereals are grown alone. Legume grains, on the other hand, are not low in protein. At the beginning of cultivation, both crops compete for nitrogen. As the cereal can utilize this better, the legume is forced to form nodules and is therefore dependent on its own production. In such associations, more nitrogen is utilized from the atmosphere than when legumes are grown alone [33]. Otherwise, competition is minimized due to the greater rooting depth of cereals compared to legumes and the different architecture of the canopy [104]. Even more specific root interactions were found in intercropping faba bean and maize. Maize root exudates lead to a deformation of the root hairs of the faba bean, which promotes the penetration of nodulating bacteria and leads to nodule formation. A higher number of nodules leads to a stronger nitrogen fixation and a higher nitrogen concentration in the soli [107,108].

**Figure 2:**
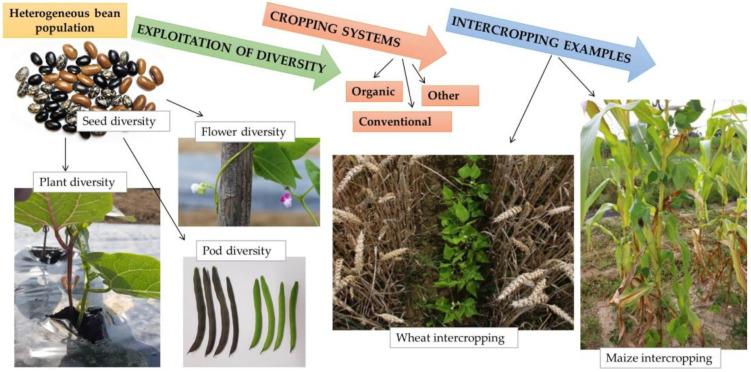
An overview of the potential of heterogeneous populations of common bean (*Phaseolus vulgaris* L.).

The common bean is a versatile legume that has been integrated into intercropping worldwide, underlining its adaptability and positive agronomic contribution (Figure 2). In Mesoamerica, the bean’s country of origin and one of the main centres of its domestication, the bean has been cultivated in multi-crop complexes for centuries. These complexes, known as La Milpa or the Three Sisters, included the climbing bean (*Phaseolus* spp.), maize (*Zea mays* L.) and squash (*Cucurbita* spp.) [4,99,109]. Each of the plants involved contributes to ecological and nutritional complementarity. Squash covers the soil, resulting in less water evaporation and weed growth, and provides a source of carbohydrates and sugars, and when the seeds are consumed, additional protein is added to the diet. Maize provides climbing support for beans and shade squash. It is also an important source of carbohydrates, proteins, and polyunsaturated oils. The third component is the bean, which with its bacterial root compounds provides nitrogen and phosphorus for growth and the overall nutritional value of this complex with proteins. All three crops are also rich in fibre, vitamins, and minerals. Such combinations are still common cropping systems in the tropics [109,110].

The La Milpa system remains and the most commonly used intercropping method for beans is maize. Such intercrops are still grown on smallholder farms in Africa, Asia, America, Spain, and Portugal [97,98,100,103]. They are particularly important and are used in regions where the risk of complete crop failure due to environmental influences is high. In intercropping, the loss of one component due to disease or extreme weather conditions can be compensated by others, which helps to mitigate yield losses. Global analyses show that plant-plant interactions are more stable in low productivity environments, suggesting potential benefits under difficult conditions [97,98,111]. Another positive aspect of beans is their ability to fix atmospheric nitrogen, which reduces the need for additional fertilisers. Bean plants initially utilise the mineral nitrogen available in the soil and seed during early growth. When this source is no longer sufficient, nitrogen fixation sets in, an energy-intensive process that leads to even better nitrogen utilisation than in monocultures. This is because the bean produces nitrogen for itself and also for the maize [33,98,111].

Maize and beans can be grown together in different ways. Climbing bean varieties are usually grown on maize, as with La Milpa, to avoid the need for support. Beans tend to twine on the maize plant when grown as an intercrop, so they grow under the maize leaves where the shading is strongest. As a result, bean yields are affected more than maize yields, but in most cases, maize is more important and the main crop [104,112]. However, to avoid competition leading to better bean growth and consequently higher nitrogen fixation rates, we need to consider using a suitable bean and maize variety to avoid competition for light or use a different cropping design [103]. Maize yield was not affected by intercropping when the bean was planted one month after maize emergence [112,113]. Another option for intercropping beans and maize is strip cropping, where two rows of beans are planted followed by two rows of maize. In this way, the advantages of the association are maintained, while at the same time, competition is reduced [104,113]. Compared to climbing varieties, bush type beans are less competitive and even more suitable for intercropping with maize. Low-growing bush beans and maize are also suitable for intercropping for silage production due to low competition and high protein, and other nutrient qualities. However, even with competition and lower yields, the yield per plot in intercropping (land equivalent ratio) is still higher than in monocropping due to the higher plant density and the growth of two different plants in the same area [103,112].

Overall, the efficiency of such mixtures depends largely on the growth habit and diversity of the crops, the planting density, the time of planting of the individual crops, the location, and the weather conditions during the growing seasons [103,114,115]. We have shown that intercropping offers numerous advantages and appears to be an important strategy for the future. However, some studies also suggest that such intercropping is not suitable and even more difficult to manage when weed infestations occur and a dicotyledonous crop is grown with a monocotyledonous crop, which reduces herbicide options [33,113]. Another dilemma is the cultivation of two crops when only one is desired. Other factors limiting the use of intercropping is the lack of mechanization options and post-harvest sorting due to the less desirable heterogeneous products [33,99,103].

## 5. Conclusions

The heterogeneity of the common bean, reflected in the different colours of the seed coat, is a multifaceted aspect influenced by environmental factors, outcrossing mechanisms and the complex interaction of numerous genes involved in the formation of the seed coat. The presence of this heterogeneity poses challenges for breeders, particularly in terms of registration and maintenance. However, it also opens up the possibility of using diversity to advantage. Landraces, which are characterized by historical and geographical influences, show the adaptability of beans to different environments. Mixtures of different bean varieties and intercropping systems in which different populations work together in the fields are proving to be strategic approaches. These systems not only utilize the inherent resilience of heterogeneity, but also increase productivity by increasing plant density and diversity in agroecosystems. To understand the complexity of common bean heterogeneity, we need to understand the intricate genetic, ecological, and breeding aspects. Incorporating and utilizing this diversity, whether through landraces or innovative agricultural practices, promises to ensure the resilience and sustainability of common bean cultivation in the face of changing challenges. Ultimately, the diversity of common bean heterogeneity is proving to be a valuable resource that offers solutions for sustainable agriculture and food security.
